# Relationship between insulin-like growth factor-1 (IGF-1) concentrations and body trait measurements and climatic factors in prepubertal goat kids

**DOI:** 10.5194/aab-62-241-2019

**Published:** 2019-05-02

**Authors:** Erkan Pehlivan

**Affiliations:** Department of Animal Science, Faculty of Agriculture, Ankara University, Ankara, 06110, Turkey

## Abstract

This study aimed to investigate relations between insulin-like growth factor-1 (IGF-1) concentrations and some body trait measurements (body weight, withers height, rump height, body length, chest depth, chest width, chest girth and cannon bone circumference) and climatic factors in prepubertal male and female White (75 % Saanen and 25 % Kilis goat) and Angora goat kids. For this purpose, blood samples were regularly taken from the vena jugularis, and body trait measurements were regularly carried out (every 15 d for 5 months) on each kid. The IGF-1 analysis on the blood serum was performed using the enzyme immunoassay (EIA) method. Climatic values and the length of the photoperiod were obtained from the Turkish State Meteorological Service for the experimental period, and the temperature–humidity index (THI) was calculated using these values. Statistical analysis showed that the IGF-1 concentrations were higher (P<0.05) in female White goat kids. Furthermore, differences in IGF-1 concentrations were found (P<0.05) between periods and between the gender groups for both the White and the Angora goat kids. Moreover, the difference between the IGF-1 concentrations between genders was higher (P<0.05) in White goat kids. Additionally, positive and significant correlations were found between IGF-1 concentrations and some body trait measurements in prepubertal kids, except for in female White goat kids. In summary, it was found that there was a significant relationship between IGF-1 concentrations and growth characteristics of the goat kids. Furthermore, IGF-1 concentrations in the goat kids were significantly influenced by climatic factors such as photoperiod, temperature and the temperature–humidity index, with the release of IGF-1 increasing due to increases in the photoperiod and the environmental temperature.

## 1 Introduction

Insulin-like growth factors (IGFs) are one of the most important compounds that influence metabolism in animals. IGFs are a family of polypeptides that carry out metabolic and mitogenic activities. The most potent mitogenic IGF peptide is insulin-like growth factor-1 (IGF-1), a basic polypeptide of 70 amino acids directed by growth hormone under normal physiological conditions (Buonomo et al., 1988). IGF-1 is mainly produced in the liver in addition to being produced by the environmental tissues such as the skin, ovary, placenta, breast and bone as autocrine/paracrine (Hashizume et al., 2000; Basturk, 2007). The bioactivity of IGF-1 is achieved by specific insulin-like growth factor binding proteins (IGFBPs) with high affinity (Obese et al., 2008).

IGF-1 plays an important role in various physiological processes such as reproduction, growth, lactation and the health of the organism (McGuire et al., 1992). IGF-1 is involved in the growth and function of almost every organ in the body (Rasouli et al., 2017). The predominant physiological effect of IGF-1 is the stimulation of postnatal body growth. In addition, IGF-1 can regulate the synthesis of whole body protein, the uptake of glucose by peripheral tissues and the regulation of lipid metabolism (Hadsell et al., 2002).

IGF-1 is secreted nonpulsatile, and the secretion level of IGF-1 is phenotypically correlated with the live weight and growth rate in cattle, pigs, sheep and chickens (Bishop et al., 1989). In addition, it has been reported that IGF-1 concentrations in farm animals are significantly affected by environmental factors (Sarko et al., 1994). As a matter of fact, Spicer et al. (1994) and Dahl et al. (1997) reported that photoperiod and IGF-1 concentration were positively correlated, Richards et al. (1995) reported a negative correlation between environmental temperature and IGF-1 concentration, Moyes et al. (2003) reported that plasma IGF-1 concentrations decreased when animals were in negative energy balance during the postpartum period, Squires (2003) reported that short-term stress reduced IGF-1 secretion, and Magistrelli et al. (2005) reported that energy and protein contents of rations are directly related to the plasma IGF-1 concentration. However, studies regarding the association of IGF-1 concentrations with growth traits and climatic factors have mainly been carried out on farm animal species other than goats. From this point on, in this study, the relationship between IGF-1 concentrations and some body trait measurements and environmental factors such as temperature and photoperiod are investigated in prepuberty period in male and female kids from two goat breeds that have different growth rates (Cengiz et al., 1995; Erol et al., 2014); this difference is due to the fact that the Angora goat is a fiber goat and the White goat is a milk goat.

## 2 Materials and methods

### 2.1 Experimental animals, location and management

This study was carried out on 13 White goat (75 % Saanen and 25 % Kilis goat) kids and 12 Angora goat kids raised in the Animal Husbandry Station (39${}^{\circ }$57${}^{\prime }$42.5${}^{\prime \prime }$ N, 32${}^{\circ }$51${}^{\prime }$56.2${}^{\prime \prime }$ E) at the Ankara University, Faculty of Agriculture, Department of Animal Science. All of the goat kids were clinically healthy. The animals were housed in shaded pens with their dams, and natural light from windows and a door was allowed to pass through to the pens. The goat kids were allowed to receive milk from their dams during the experimental period, and from 2 weeks old they were they were fed concentrate feed and alfalfa hay ad libitum. Experimental feeds were collected during the experimental period and analyzed for chemical composition. Dry matter, crude protein, crude fiber, crude ash and crude fat in feeds were analyzed according to the AOAC Official Methods of Analysis (AOAC, 2000). Neutral detergent fibre (NDF) and acid detergent fibre (ADF) were measured according to the methods described by Van Soest et al. (1991). The metabolizable energy (ME) of forage and concentrate mix was calculated according to the Turkish Standards Institute (TSE, 1991). The concentrated feed composition and ME was as follows: dry matter (DM) comprised 91 %, crude protein (CP) comprised 18.05 %, crude fiber (CF) comprised 8 %, crude ash comprised 7.5 % and ether extract (EE) comprised 3.5 %; the ME was 2615 Kcal kg${}^{-1}$. The alfalfa composition and ME was as follows: DM comprised 91.5 %, CP comprised 13.0 %, CF comprised 34.0 %, crude ash comprised 8.29 %, EE comprised 1.00 %, ADF comprised 8.0 %, NDF comprised 49.0 %; the ME was 2000.22 kcal kg${}^{-1}$. Fresh water was always available to the goat kids. Management of experimental goat kids did not interfere with the general operation of the station, and the study was conducted within standard ethical norms. Some properties of experimental goat kids are shown in Table 1.

**Table 1:** Some properties of the experimental goat kids.

Breed	Gender	n	Average birth	Average age at the beginning	Average live weights at the
			weight (kg)	of experiment (days)	beginning of experiment (kg)
White goat (n=13)	Male	7	3.06	14	6.53
	Female	6	3.10	15	6.51
Angora goat (n=12)	Male	7	2.91	15	5.51
	Female	5	2.50	14	4.39

### 2.2 Blood collection and IGF-1 analysis

During the experimental period, the first samples (Period 1) were taken 14–15 d after birth, and samples (periods 2–10) were the taken at 15 d intervals for 5 months. On sampling days, blood samples were regularly taken from the vena jugularis of each goat kid using vacuum containers without anticoagulant (VACUETTE^®^ TUBE 8 mL Z Serum Sep Clot Activator). The blood samples were centrifuged at 4000×g for 5 min, and the serum was stored at -20 ${}^{\circ }$C until the analysis was carried out. IGF-1 concentrations were determined in the blood serum using a commercial ELISA kit (Fine Test Goat IGF-1 Cat. no. EG0002). The intra- and inter-assay coefficients of variation were <8 % and <10 %, respectively. The least detectable concentration was 14 ng mL${}^{-1}$.

### 2.3 Measurement of body traits

With respect to body trait measurements, the body weight (BW), withers height (WH), rump height (RH), body length (BL), chest depth (CD), chest width (CW), chest girth (CG) and cannon bone circumference (CBC) of each kid were regularly measured on the same sampling days. BW was measured using a commercial hanging scale (±10 g). A measuring tape was used to measure the height, length, depth and width of the goat kids, whereas other body measurements were taken using a flexible tape measure. All measurements were taken by the same operator and followed the methodology of Herrera et al. (1996).

### 2.4 Climatic values, the temperature–humidity index (THI) and the photoperiod

Climatic values and the photoperiod were obtained from the Turkish State Meteorological Service (Anonymous, 2016) in order to estimate the severity of heat stress during the experimental period. The temperature–humidity index (THI) was calculated using the following equation, reported by Marai et al. (2001) for sheep and goats:

THI = db ${}^{\circ }$C - {(0.31 - 0.31 RH/100) (db ${}^{\circ }$C - 14.4)},

where db ${}^{\circ }$C is the dry bulb temperature (${}^{\circ }$C) and RH is the relative humidity (RH %)/100. The values obtained indicate the following: < 22.2 signifies the absence of heat stress; 22.2 to < 23.3 represents moderate heat stress: 23.3 to <25.6 represents severe heat stress and 25.6 and above represents extreme severe heat stress

(Marai et al., 2007).

In addition, the average climatic values, the THI index values and the photoperiod lengths are shown in Table 2.

**Table 2:** Average climatic values, the temperature–humidity index (THI) and the length of the photoperiod during the experimental period.

Periods${}^{*}$	Average	Average	THI	Photoperiod
	temperature	humidity		(hour:min)
	(${}^{\circ }$C)	(%)		
1	10.8	51.0	11.4	13:18
2	18.5	31.5	17.6	13:50
3	13.9	45.9	14.0	14:20
4	15.0	58.2	14.9	14:43
5	17.1	78.5	17.0	14:58
6	18.3	65.6	17.9	15:01
7	23.7	50.1	22.3	14:52
8	27.4	35.6	24.8	14:31
9	26.1	43.6	24.1	14:03
10	24.2	62.7	23.1	13:33

${}^{*}$ The first samples (Period 1) were taken 14–15 d after birth, and the other samples (periods 2–10) were the taken at 15 d intervals.

### 2.5 Statistical analysis

Data were analyzed by a mixed model in SAS (2017) using repeated measurement analysis with breed, gender and period as fixed effects and the live weight of the animal at the beginning of the experiment as the covariate. IGF-1 concentration differences within and between groups and their interactions were evaluated. Multiple comparisons were made using a Tukey test if significance was indicated by the analysis of variance. Correlation coefficients (Pearson) between IGF-1 concentrations and some body trait measurements were calculated according to the CORR procedure in SAS (2017). Results are shown as the mean ± standard error ($\bar{X}\pm S\bar{x}$), and the significance level was set at α=0.05.

## 3 Results

### 3.1 Climatic values during the experimental period

**Figure 1:**
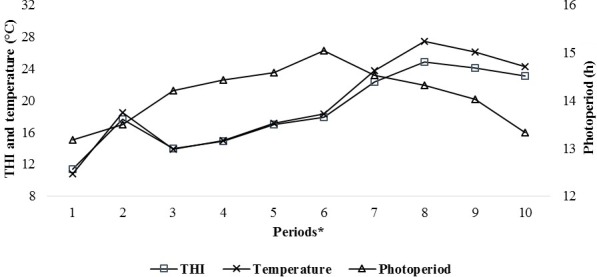
Changes in the average temperature, the temperature–humidity index (THI) and the photoperiod during the experimental periods. ${}^{*}$ The first samples (Period 1) were taken 14–15 d after birth, and the other samples (periods 2–10) were then carried out at 15 d intervals.

Patterns of climatic values during the experiment period are shown in Fig. 1. As seen from Fig. 1, the photoperiod values increased until Period 6, the THI and temperature values increased until Period 8 and then they all gradually decreased thereafter. It has been determined that the experimental kids were heat stressed during the last periods (from Period 6 to Period 10) of the experiment (Table 2).

### 3.2 IGF-1 concentrations in prepubertal goat kids

**Figure 2:**
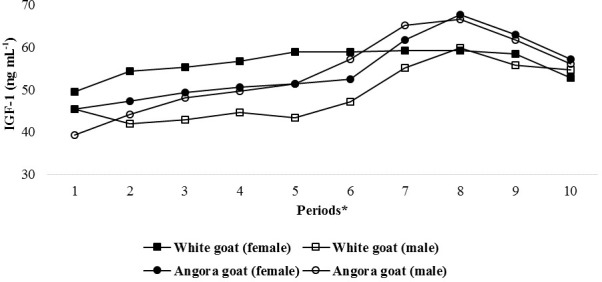
Changes in IGF-1 concentrations of kids during the experimental periods. ${}^{*}$ The first samples (Period 1) were taken 14–15 d after birth, and the other samples (periods 2–10) were then carried out at 15 d intervals.

IGF-1 concentrations in experimental kids progressively increased until Period 8 and then progressively decreased until end of the experiment (Fig. 2); the differences in the IGF-1 concentrations between the periods were found to be statistically significant (P<0.05; Table 3). Significant differences were found (P<0.05) with respect to IGF-1 concentrations between female and male kids in White goat kids, whereas no significant differences between female and male kids were observed in Angora goat kids (regarding IGF-1 concentrations). Differences between the same genders from different breeds were also found to be statistically important (P<0.05).

**Table 3:** Least squares means and standard errors ($\bar{X}\pm S\bar{x}$) of IGF-1 concentrations in White goat and Angora goat kids.

Periods*	White goat		Angora goat	
	Female (n=6)	Male (n=7)	Female (n=5)	Male (n=7)
1	$50.98\pm 1.95^{\mathrm{XaA}}$	$46.94\pm 1.81^{\mathrm{XabA}}$	$42.46\pm 2.25^{\mathrm{aB}}$	$38.59\pm 1.77^{\mathrm{aB}}$
2	$55.78\pm 2.49^{\mathrm{XbA}}$	$43.50\pm 2.31^{\mathrm{YaA}}$	$44.32\pm 2.82^{\mathrm{aB}}$	$43.46\pm 2.28^{\mathrm{bA}}$
3	$56.78\pm 3.30^{\mathrm{XabA}}$	$44.37\pm 3.06^{\mathrm{YaA}}$	$46.33\pm 3.69^{\mathrm{abA}}$	$47.45\pm 3.04^{\mathrm{cA}}$
4	$58.15\pm 3.23^{\mathrm{XbA}}$	$46.07\pm 3.00^{\mathrm{YaA}}$	$47.65\pm 3.61^{\mathrm{abB}}$	$48.91\pm 2.97^{\mathrm{cdA}}$
5	$60.36\pm 3.26^{\mathrm{XbA}}$	$44.81\pm 3.02^{\mathrm{YaA}}$	$48.34\pm 3.64^{\mathrm{abB}}$	$50.63\pm 3.00^{\mathrm{cdA}}$
6	$60.38\pm 4.21^{\mathrm{XabA}}$	$48.68\pm 3.90^{\mathrm{XabcA}}$	$49.46\pm 4.67^{\mathrm{abA}}$	$56.49\pm 3.88^{\mathrm{efA}}$
7	$60.73\pm 4.63^{\mathrm{XabA}}$	$56.66\pm 4.29^{\mathrm{XdA}}$	$58.75\pm 5.12^{\mathrm{cdA}}$	$64.56\pm 4.27^{\mathrm{gA}}$
8	$60.69\pm 3.94^{\mathrm{XbA}}$	$61.34\pm 3.65^{\mathrm{XdA}}$	$64.58\pm 4.37^{\mathrm{dA}}$	$65.93\pm 3.63^{\mathrm{gA}}$
9	$59.82\pm 3.84^{\mathrm{XbA}}$	$57.27\pm 3.56^{\mathrm{XdA}}$	$59.93\pm 4.27^{\mathrm{cdA}}$	$61.02\pm 3.54^{\mathrm{fgA}}$
10	$54.30\pm 3.65^{\mathrm{XabA}}$	$56.14\pm 3.39^{\mathrm{XcdA}}$	$54.21\pm 4.07^{\mathrm{bcA}}$	$55.35\pm 3.37^{\mathrm{deA}}$

Notes: a, b, c, d, e, f, g: Means in the same column with different superscripts differ significantly at p<0.05. X, Y: Means in the same row with different superscripts differ significantly at p<0.05 (in the comparison of the genders within breeds). A, B: Means in the same row with different superscripts differ significantly at p<0.05 (in the comparison of the same gender between breeds). ${}^{*}$ The first samples (Period 1) were taken 14–15 d after birth, and the other samples (periods 2–10) were then carried out at 15 d intervals.

### 3.3 Some body trait measures in prepubertal goat kids

Periodic mean values of some body trait measures for White and Angora goat kids are shown in Tables 4 and 5, respectively. An almost linear increase was observed in all of the body trait measures in all of goat kids examined during the experimental period. When the body weights of kids at the end of the experiment were compared with their birth weights, it was found that White goat kids grew more than Angora goat kids, and male kids grew more than female kids.

**Table 4:** Periodic mean values ($\bar{X}\pm S\bar{x}$) of some body trait measures in White goat kids.

Periods${}^{*}$	Gender	BW (kg)	WH (cm)	RH (cm)	BL (cm)	CD (cm)	CW (cm)	CG (cm)	CBC (cm)
1	F	6.52±0.75	34.83±0.87	34.50±0.92	35.17±0.95	14.50±0.67	9.50±0.43	43.00±1.61	6.30±0.16
	M	6.53±0.50	34.86±0.70	35.14±0.86	36.14±0.83	13.71±0.18	9.14±0.26	43.00±1.25	6.79±0.15
2	F	8.84±0.85	38.50±1.63	40.17±1.94	38.67±1.58	14.50±0.43	10.17±0.54	49.17±2.08	6.38±0.12
	M	7.86±0.84	40.57±0.57	40.86±0.59	37.86±1.50	14.57±0.48	8.43±0.72	46.29±2.00	6.54±0.15
3	F	11.37±0.85	44.00±1.44	44.50±1.73	42.00±1.21	15.83±0.40	11.00±0.52	53.00±1.42	6.58±0.14
	M	10.41±0.97	44.71±1.04	45.00±1.07	41.00±1.66	15.57±0.48	9.86±0.70	50.93±2.21	6.73±0.20
4	F	13.76±0.94	49.83±1.17	49.50±1.45	45.50±1.34	18.17±0.48	12.58±0.58	54.33±2.51	6.78±0.12
	M	12.43±1.46	49.14±1.68	49.57±2.06	44.57±2.06	16.93±0.64	11.21±0.71	53.57±2.43	6.86±0.25
5	F	16.64±0.75	51.00±0.68	51.17±1.08	49.50±0.76	20.00±0.37	12.83±0.48	60.25±1.35	7.08±0.07
	M	15.17±1.87	52.86±1.75	50.86±1.75	48.14±2.19	19.36±0.82	11.86±0.63	57.00±2.09	7.07±0.27
6	F	17.78±0.77	51.83±0.54	52.00±0.82	50.83±0.60	20.67±0.42	13.58±0.33	60.67±1.44	7.20±0.10
	M	17.54±1.99	53.29±1.67	52.00±1.50	49.14±2.18	21.00±0.93	12.79±0.60	57.93±2.08	7.44±0.30
7	F	19.04±0.81	54.00±0.37	55.33±0.21	50.83±0.40	21.83±0.87	12.83±0.40	63.17±1.35	7.37±0.13
	M	19.29±2.16	55.43±2.19	56.14±1.83	50.00±2.23	21.43±0.72	12.43±0.81	61.86±2.77	7.49±0.32
8	F	19.61±1.08	55.67±1.20	57.00±0.82	52.50±0.76	21.50±0.56	12.83±0.48	64.33±1.50	7.27±0.13
	M	20.60±2.40	57.57±2.26	58.29±1.92	53.14±2.67	22.00±0.93	12.86±0.70	62.00±2.96	7.31±0.43
9	F	20.94±1.14	56.67±0.80	59.00±0.86	52.33±1.15	22.33±0.33	13.17±0.48	65.92±1.17	7.27±0.15
	M	21.92±2.68	58.57±2.00	59.00±1.98	55.00±3.54	22.43±0.95	12.43±0.81	65.64±3.76	7.39±0.47
10	F	22.64±1.08	57.33±0.84	60.50±0.96	55.17±1.30	22.50±0.34	13.17±0.83	66.67±1.82	7.50±0.12
	M	23.42±2.90	59.14±2.19	60.14±2.02	54.12±2.42	22.71±0.97	13.29±0.99	66.29±2.90	7.69±0.33

F: female; M: male; BW: body weight; WH: withers height; RH: rump height; BL: body length; CD: chest depth; CW: chest width; CG: chest girth; CBC: cannon bone circumference. ${}^{*}$ The first samples (Period 1) were taken 14–15 d after birth, and the other samples (periods 2–10) were then carried out at 15 d intervals.

**Table 5:** Periodic mean values ($\bar{X}\pm S\bar{x}$) of some body trait measures in Angora goat kids.

Periods${}^{*}$	Gender	BW (kg)	WH (cm)	RH (cm)	BL (cm)	CD (cm)	CW (cm)	CG (cm)	CBC (cm)
1	F	4.40±0.32	29.40±0.93	29.80±0.73	29.60±0.68	12.00±0.55	7.00±0.45	37.00±1.26	5.42±0.13
	M	5.51±0.44	32.57±0.95	32.86±0.94	32.00±0.72	12.86±0.51	7.86±0.26	39.79±0.98	6.11±0.26
2	F	6.17±0.48	34.00±1.49	34.40±1.12	34.80±0.66	11.80±0.49	8.40±0.40	41.00±1.59	5.44±0.15
	M	7.49±0.62	37.29±1.08	38.00±1.23	36.00±0.85	13.86±0.46	8.71±0.47	44.64±1.16	6.20±0.17
3	F	8.02±0.69	38.80±0.66	38.80±0.80	37.40±0.81	13.60±0.60	9.40±0.40	46.30±1.58	5.92±0.14
	M	9.73±0.91	42.57±1.21	42.71±1.23	39.00±0.98	15.29±0.52	10.00±0.44	50.07±1.37	6.40±0.21
4	F	10.06±0.96	43.60±0.51	43.60±0.51	40.00±1.34	15.60±0.93	10.80±0.49	49.40±2.20	5.88±0.21
	M	12.90±0.89	48.00±1.29	48.00±1.27	42.00±1.11	17.07±0.71	11.64±0.37	53.86±1.35	6.66±0.18
5	F	11.46±1.12	46.40±1.36	46.40±1.36	42.80±1.36	15.50±0.59	11.10±0.33	51.80±2.31	6.20±0.31
	M	15.00±1.08	49.86±1.53	51.43±1.31	46.71±1.17	18.43±0.69	12.14±0.32	56.71±1.20	6.93±0.28
6	F	12.72±1.34	47.20±1.53	47.60±1.50	44.40±1.69	16.90±0.78	11.90±0.51	51.90±2.33	6.36±0.30
	M	16.24±1.26	50.86±1.50	52.00±1.51	47.29±1.23	19.86±0.51	12.79±0.43	57.07±1.21	7.34±0.28
7	F	13.36±1.28	48.40±1.86	49.60±1.99	47.20±0.37	18.40±0.68	12.10±0.68	55.60±2.50	6.16±0.12
	M	17.29±1.41	53.57±2.01	54.57±1.60	48.71±0.97	20.29±0.47	12.43±0.43	59.57±1.56	6.87±0.23
8	F	14.31±1.46	48.80±1.77	50.20±1.83	46.80±1.98	17.60±0.93	11.80±0.80	57.20±2.71	6.06±0.34
	M	18.46±1.69	54.00±2.43	55.00±2.17	49.71±1.52	20.14±0.63	12.43±0.43	61.07±1.46	6.91±0.28
9	F	14.44±1.48	49.40±1.40	50.60±1.54	48.00±2.07	17.40±1.03	11.40±0.51	55.60±1.94	6.16±0.31
	M	19.17±1.91	54.29±2.83	56.57±2.47	51.29±2.02	20.86±0.77	13.14±0.67	62.29±1.87	7.07±0.25
10	F	14.99±1.65	49.80±2.22	51.40±2.40	47.40±1.91	19.00±0.77	12.80±0.66	57.60±3.04	6.14±0.17
	M	20.74±2.02	54.71±2.33	57.29±2.38	52.29±1.80	21.00±0.69	13.29±0.52	63.86±1.74	7.03±1.39

F: female; M: male; BW: body weight; WH: withers height; RH: rump height; BL: body length; CD: chest depth; CW: chest width; CG: chest girth; CBC: cannon bone circumference. ${}^{*}$ The first samples (Period 1) were taken 14–15 d after birth, and the other samples (periods 2–10) were then carried out at 15 d intervals.

### 3.4 Correlations between IGF-1 concentrations and some body trait measures in prepubertal goat kids

Phenotypic correlations between IGF-1 and some body trait measures in White goat and Angora goat kids are shown in Table 6. Correlations between IGF-1 and some body trait measures were not found to be statistically significant (P>0.05) in female White goat kids, whereas these correlations (except CBC) were found to be statistically significant (P<0.01) in male White goat kids. In Angora goat kids, IGF-1 concentrations were correlated with BW (P<0.01), WH (P<0.01), RH (P<0.01), BL (P<0.01), CD (P<0.01), CW (P<0.01), CG (P<0.01) and CBC (for females: P<0.01; for males P<0.05).

**Table 6:** Correlation coefficients between IGF-1 and some body trait measures in prepubertal goat kids.

Breeds	Gender		BW	WH	RH	BL	CD	CW	CG	CBC
White goat kids	F	IGF-1	-0.039	0.134	0.059	0.011	0.092	-0.187	-0.028	-0.085
	M	IGF-1	0.371${}^{**}$	0.332${}^{**}$	0.367${}^{**}$	0.330${}^{**}$	0.400${}^{**}$	0.301${}^{*}$	0.318${}^{**}$	0.171
Angora goat kids	F	IGF-1	0.700${}^{**}$	0.648${}^{**}$	0.678${}^{**}$	0.737${}^{**}$	0.680${}^{**}$	0.521${}^{**}$	0.691${}^{**}$	0.448${}^{**}$
	M	IGF-1	0.541${}^{**}$	0.590${}^{**}$	0.620${}^{**}$	0.634${}^{**}$	0.593${}^{**}$	0.590${}^{**}$	0.606${}^{**}$	0.246${}^{*}$

${}^{**}$ P<0.01, ${}^{*}$ P<0.05. F: female; M: male; BW: body weight; WH: withers height; RH: rump height; BL: body length; CD: chest depth; CW: chest width; CG: chest girth; CBC: cannon bone circumference.

## 4 Discussion

This study was carried out from mid-April to mid-August, which comprised a range of seasonal variations regarding factors such as the THI, the temperature and the photoperiod that could affect the IGF-1 concentrations of the experimental goat kids. Thus, the effects of climatic factors such as temperature, heat stress and photoperiod on IGF-1 concentrations of experimental kids may have been confounded with one another. Because the experiment was not carried out under controlled photoperiod/temperature conditions, it was not clear which climatic factors more heavily impacted the IGF-1 concentrations of the prepubertal kids. However, in this study, the pattern of IGF-1 concentrations in the kids was similar to the change in the photoperiod during the experimental period (Fig. 2). It has also been previously reported that IGF-1 concentrations in farm animals are significantly affected by environmental factors (Sarko et al., 1994). In many previous studies, especially those conducted on ruminants, it is suggested that seasonal changes in IGF-1 concentrations are mainly driven by photoperiod (Dahl et al., 2000). In addition, it has been reported that the photoperiod has a significant effect on IGF-1 concentrations in goats (Flores et al., 2015, 2018; Hernández et al., 2016). Similarly, in a study carried out on male red deer, it was reported that melatonin implantation, which had “short-day effects”, suppressed IGF-1 release (Suttie et al., 1992). Another study on cattle reported that the IGF-1 levels of cattle in a 16 h light per day group for 4 months were considerably higher than those in an 8 h light per day group (Spicer et al., 1994). Furthermore, in studies investigating the effect of environmental temperature on IGF-1 concentrations in farm animals, Richards et al. (1995) found a negative correlation between environmental temperature and IGF-1 concentrations, and Aggarwal and Upadhyay (2013) reported that IGF-1 concentrations in their study decreased during the summer months. Similarly, in this study, it can be seen that IGF-1 concentrations of kids started to decrease during periods of high temperature and heat stress (Figs. 1 and 2).

As seen from Tables 4 and 5, all body trait measures increased linearly as the kids grew. However, there were differences both between breeds and between genders in terms of the growth rate. It is known that IGF-1 concentrations show a positive correlation with skeletal development, protein accumulation and growth rate in farm animals (Bishop et al., 1989). Thus, positive correlations between IGF-1 concentrations and live weight and weight gain have been reported in farm animal species such as cattle, pigs, sheep and poultry (Bishop et al., 1989; Davis and Simmen, 1997). In this study, statistically significant positive correlations were also found between IGF-1 concentrations and some body trait measures in kids, except in female White goat kids (Table 6). The presence of positive and significant correlations between IGF-1 and growth traits is an expected outcome due to the growth and development of prepubertal goat kids; however, these correlations may be negative in adult goats. As a matter of fact, it has been reported that there is a negative correlation between IGF-1 concentrations and body mass in 36 mammalian species, and that high IGF-1 levels allow for cancer growth and thus shorten the animal's life span (Stuart and Page, 2010). However, this result contrasts with the reported negative correlations (r=-0.56) between IGF-1 concentrations and live weight in Angora goat kids (Acuti et al., 2009). In summary, the findings obtained from this study are generally compatible with studies on other mammalian species.

## 5 Conclusion

Results of this study indicate that there is a significant relationship between IGF-1 concentrations and growth characteristics of goat kids. IGF-1 releases in prepubertal goat kids increased due to increases in the photoperiod and environmental temperature, and statistically significant positive correlations between IGF-1 concentrations and some body trait measures were also found in prepubertal kids.

## Data Availability

The data sets can be taken from the figures/tables or are available upon request from the corresponding author.
